# Annotations

**Published:** 1903-02-21

**Authors:** 


### ANNOTATIONS.

### "Knife and Fork" Surgery.

IT has been said, not inaptly, that the skin is the surgeon's great enemy, for it is difficult to sterilise the skin of the hands so effectually as to do away with all risk of infecting the wound during an operation. The difficulty lies not so much in sterilising the surface, which, by the exercise of care, can generally be effected, as in getting at the deeper portion of the skin from the glands in which under the influence of heat and moisture, swarms of microbes are said to be discharged. Many plans have been devised with the object of obviating, or at least, of lessening this risk. To those who practise Listerian methods the affair is comparatively simple: a basin containing an antiseptic is kept at hand, and as often as may be desirable in the course of the operation the hands are soaked in this fluid. To the modern "aseptic" surgeon, however, all this is anathema; hence the introduction of gloves, either of rubber or cotton, which can be sterilised. These are good but have their disadvantages, especially in that they interfere with the sense of touch. One distinguished English surgeon, although making all his assistants wear gloves, operates with bare hands, for the sake of retaining his tactile sensibility. Another method of preventing hand-infection of the area of operation, a method which we venture to speak of as "knife and fork" surgery has lately been described by Kœnig at Berlin. According to this an attempt is made to perform the operation without ever touching the tissues, much as we eat our meals by aid of forks and spoons instead of clawing our food. By practice with suitably devised metal instruments, which of course are capable of complete sterilisation, he has found that he can get through very many operations without ever touching the wound. Even in abdominal cases, in many of which it is often impossible to avoid introducing the hand, he carries his instrumental method to its extremest limit. In operating on cases of appendicitis during the quiescent interval he has been able to do the entire operation with instruments. This is a method which, while it has its advantages, has also its limitations. One can hardly doubt, however, that the surgeon often does things with his fingers which, by aid of a little practice, could be done just as well with an instrument, and if we are to accept as correct all that is said about hand-infection it would often be well, in suitable cases, to adopt the "knife and fork" method of surgery.

### The Period of Infectivity in Scarlet Fever.

THERE are few more awkward positions than that of the medical man who has to certify in regard to a convalescent from scarlet fever that all danger of his conveying infection has passed away, for, unfortunately, he has no definite criterion by which the question can be decided. At a recent meeting of the Society of Medical Officers of Health a paper was read by Dr. W. A. Bond, M.O.H. for Holborn, in which various points bearing upon this question were fully discussed. Let us take first the very important matter of desquamation. Dr. Bond's remarks and quotations show how largely professional opinion has veered round from the view that desquamations after scarlet fever is essentially and inherently infective, a view which is still the cause of an enormous amount of probably unnecessary expenditure by the Metropolitan Asylums Board, from whose hospitals, it is stated, patients are not as a general rule discharged before every particle of desquamating cuticle has been shed. This brings us to the question of a time test. Till the last year or two the scarlet fever patients at the Asylum Board's Hospital have been detained on an average more than ten weeks. Yet this does not put a stop to "return" cases, which, it seems from Dr. Simpson's report, are not due to premature discharge, are not to be lessened by long detention in hospital, and bear little relationship to the duration of treatment. If an average detention of nearly ten weeks, extending in some cases to a much longer period, fails to secure non-infectivity on discharge the "time test" evidently fails us entirely. What then is to be the criterion? It seems very probable that the mucous membrane of the mouth and tonsils is the principal source of infection in scarlet fever. Unfortunately, this does not furnish us with any reliable indication as to whether or not any individual case is still infective. It does, however, give us a clue to the means to be adopted for preventing prolonged infectivity. Throat and mouth disinfection, with solutions of potassium permanganate or liquor chlori, or other suitable solution, should be practised as a matter of routine. Moreover, in view of the fact that when a patient has once become immune he may receive the infection afresh and carry it about in his throat without doing himself any harm, but vastly to the injury of those he comes in contact with, it is evidently desirable that after discharge from hospital a certain quarantine should be undergone before mixing freely with susceptibles.

### "Appendicitis" and the "New English Dictionary."

MUCH has been written concerning the barbaric word "appendicitis," which medical men, and particularly those of our American cousinship, have made familiar both to the profession and the public. The following note, by the distinguished editor of the "New English Dictionary," which recently appeared in *Notes and Queries*, is of particular interest in regard to the history of "appendicitis," as well as the use of such a term. Dr. Murray writes:—"When the portion of the dictionary dealing with *app* was written in 1883, we had before us a single reference, from a recent medical source, for this word. As words in *itis* are not (in origin) English in form, but Græco Latin, and thus do not come within the scope of an English dictionary, unless, like *bronchitis*, they happen to be in English use, I referred our quotation for *appendicitis* to a well-known distinguished medical professor. . . . His answer was that *appendicitis* was a name recently given to a very obscure and rare disease; the term was purely technical or professional, and had even less claim to inclusion in an English dictionary than hundreds of other Latin or Latinised Greek terms of which the medical lexicons are full, and which no one thinks of as English." We could have wished that Dr. Murray had told us who was the "well-known distinguished medical professor."

